# Comparison of Surrogate Markers of the Type I Interferon Response and Their Ability to Mirror Disease Activity in Systemic Lupus Erythematosus

**DOI:** 10.3389/fimmu.2021.688753

**Published:** 2021-06-30

**Authors:** Helena Enocsson, Jonas Wetterö, Maija-Leena Eloranta, Birgitta Gullstrand, Cecilia Svanberg, Marie Larsson, Anders A. Bengtsson, Lars Rönnblom, Christopher Sjöwall

**Affiliations:** ^1^ Department of Biomedical and Clinical Sciences, Division of Inflammation and Infection, Linköping University, Linköping, Sweden; ^2^ Department of Medical Sciences, Rheumatology, Uppsala University, Uppsala, Sweden; ^3^ Department of Clinical Sciences Lund, Division of Rheumatology, Lund University, Lund, Sweden; ^4^ Department of Biomedical and Clinical Sciences, Division of Molecular Medicine and Virology, Linköping University, Linköping, Sweden

**Keywords:** SLE, lupus, biomarker, disease activity, interferon, chemokine, galectin, TNF

## Abstract

**Objectives:**

Type I interferons (IFNs) are central and reflective of disease activity in systemic lupus erythematosus (SLE). However, IFN-α levels are notoriously difficult to measure and the type I IFN gene signature (IGS) is not yet available in clinical routine. This study evaluates galectin-9 and an array of chemokines/cytokines in their potential as surrogate markers of type I IFN and/or SLE disease activity.

**Methods:**

Healthy controls and well-characterized Swedish SLE patients from two cross-sectional cohorts (*n*=181; *n*=59) were included, and a subgroup (*n*=21) was longitudinally followed. Chemokine/cytokine responses in immune complex triggered IFN-α activity was studied in healthy donor peripheral blood mononuclear cells (PBMC). Levels of chemokines/cytokines and galectin-9 were measured by immunoassays. Gene expression was quantified by qPCR.

**Results:**

The IGS was significantly (p<0.01) correlated with galectin-9 (rho=0.54) and CXCL10 (rho=0.37) levels whereas serum IFN-α correlated with galectin-9 (rho=0.36), CXCL10 (rho=0.39), CCL19 (rho=0.26) and CCL2 (rho=0.19). The strongest correlation was observed between galectin-9 and TNF (rho=0.56). IFN-α and disease activity (SLEDAI-2K) were correlated (rho=0.20) at cross-sectional analysis, but no significant associations were found between SLEDAI-2K and galectin-9 or chemokines. Several inflammatory mediators increased at disease exacerbation although CCL19, CXCL11, CXCL10, IL-10 and IL-1 receptor antagonist were most pronounced. Immune complex-stimulation of PBMC increased the production of CCL2, CXCL8 and TNF.

**Conclusion:**

Galectin-9 and CXCL10 were associated with type I IFN in SLE but correlated stronger with TNF. None of the investigated biomarkers showed a convincing association with disease activity, although CXCL10 and CCL19 performed best in this regard.

## Introduction

Patients affected by systemic lupus erythematosus (SLE) may present with a broad variety of symptoms or manifestations and episodes of raised disease activity may be followed by periods of remission. Activation of the type I interferon (IFN) system is salient for the initiation of autoimmunity and constitutes a driver of further SLE pathogenesis where immune complexes (ICs) of autoantibodies directed against nuclear constituents are deposited in various tissues (1). Nucleic acid containing ICs are considered a key inducer of type I IFNs in SLE. Fcγ receptor IIa facilitates intracellular uptake of ICs and subsequent activation of TLR-7 *(e.g.* by small nuclear ribonucleoproteins; snRNP) or TLR-9 (by DNA), primarily in plasmacytoid dendritic cells (pDCs) (1, 2). The central role of type I IFNs, including IFN-α, in SLE has been thoroughly investigated. At a given time-point, approximately 50% of patients have upregulated type I IFN induced genes – the type I IFN gene signature (IGS) (3). However, the IGS is not a standardised measurement and is not yet used clinically to evaluate disease activity in SLE.

As circulating IFN-α appears to correlate with SLE disease activity, and possibly associate with certain disease manifestations (4–6), there is great interest to attain a readily accessible and standardised method to quantify IFN-α and introduce such assays into clinical practice (7). Today, disease activity in SLE is mainly assessed by indexes of which some combine clinical and laboratory findings (8). Circulating levels of IFN-α in SLE are often below the limit of detection in immunoassays despite the presence of IGS, and surrogate markers of the type I IFN activity and/or disease activity are therefore warranted. Especially given the encouraging results of the phase 3 trial on the anti-IFN α/β receptor anifrolumab in patients with active SLE, such assays could guide the selection of patients likely to benefit from IFN-blocking treatment (9).

Previous studies have indicated type I IFN-regulated chemokines (*e.g.* CXCL10, CCL2 and CCL19) and other chemokines (CXCL8/IL-8) as potential biomarkers for disease activity in SLE (10–15). Two recent studies proposed galectin-9 as a surrogate marker of type I IFN in SLE with potential to be used in clinical routine for disease activity monitoring (16, 17).

Galectin-9 is a ubiquitously expressed glycan binding protein with carbohydrate recognition domains. It is produced by a variety of cell types and exerts several functions of relevance in autoimmunity, *e.g.* it is involved in cell adhesion and migration, induces maturation and TNF production of dendritic cells, drives expansion of regulatory T cells as well as modulating the Th1 and Th17 immunity through induction of apoptosis (18). High expression of galectin-9 is found in the liver and plasma levels increase upon liver damage caused by viral or autoimmune hepatitis (19).

The present study was undertaken to investigate galectin-9 and a broad set of chemokines/cytokines regarding their ability to reliably reflect the type I IFN response and SLE disease activity.

## Materials and Methods

### Patients and Controls

**Figure 1:**
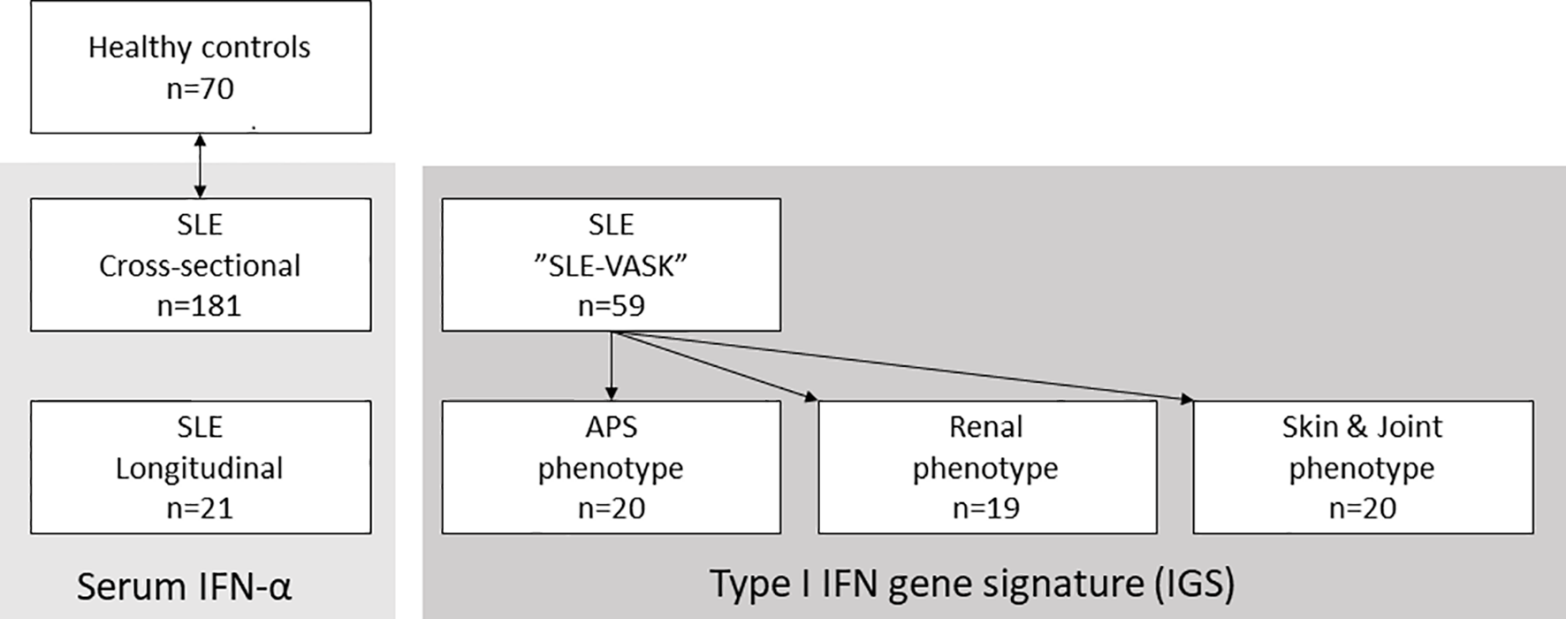
Patient groups and controls. Schematic illustration of the three major patient groups of systemic lupus erythematosus (SLE) and the healthy controls matched with the 181 patients for cross-sectional analysis. Serum interferon-α (IFN-α) was measured in the 181 cross-sectional samples and in samples from 21 longitudinally followed patients. The type I IFN gene signature (IGS) was measured in patients participating in the cross-sectional “SLE-VASK”.

All included SLE patients were participants of a prospectively enrolling regional quality register based at the University Hospital in Linköping (20). The included patients (**Table 1** and **Figure 1**), were classified with SLE based on the 1982 American College of Rheumatology (ACR-82) (21) and/or the 2012 Systemic Lupus International Collaborating Clinics (SLICC-12) criteria (22).

**Table 1:** Basic characteristics of patients with SLE divided in the 3 groups.

	SLE: Cross-sectional *n=181 Mean (range or %)*	SLE: “SLE-VASK” *n=59 Mean (range or %)*	SLE: Longitudinal *n=21 Mean (range or %)*
Age at sampling (years)	50.4 (18–88)	43.2 (23–63)	44.5 (20–75)^a^
Female gender (*n*)	162 (89.5%)	51 (86.4%)	16 (76.2%)
Disease duration (years)	12.0 (0–45)	12.1 (1–35)	10.8 (0–28)^a^
SLEDAI-2K	2.2 (0–16)	2.0 (0-10)	Not recorded^a^
cSLEDAI	1.4 (0–12)	0.5 (0–8)	0^a^
PGA	0.4 (0–4)	0.2 (0–2)	0^a^
Daily prednisolone dose (mg)	4.8 (0–60)	2.5 (0–15)	4.6 (0–15)^a^
Number of fulfilled ACR-82 criteria (*n*)	4.7 (3–9)	5.0 (3–9)	5.1 (4–8)
Daily use of HQ	90 (49.7%)	53 (89.8%)	14 (66.7%)
Fulfils ACR-82 criteria (*n*)	154 (85.1%)	55 (93.2%)	21 (100%)
Fulfils SLICC-12 criteria (*n*)	176 (97.2%)	58 (98.3%)	21 (100%)
Fulfils ACR-82 and/or SLICC-12 (*n*)	181 (100%)	59 (100%)	21 (100%)
*Fulfilled ACR-82 criteria:*			
1. Malar rash	84 (46.4%)	24 (40.7%)	7 (33.3%)
2. Discoid lupus	31 (17.1%)	2 (3.4%)	2 (9.5%)
3. Photosensitivity	97 (53.6%)	27 (45.8%)	9 (42.9%)
4. Oral ulcers	17 (9.4%)	13 (22.0%)	3 (14.3%)
5. Arthritis	139 (76.8%)	46 (78.9%)	18 (85.7%)
6. Serositis	72 (39.8%)	23 (39.0%)	9 (42.9%)
7. Renal disorder	41 (22.7%)	19 (32.2%)	14 (66.7%)
8. Neurological disorder	11 (6.1%)	6 (10.2%)	0 (0%)
9. Haematological disorder	100 (55.2%)	39 (66.1%)	12 (51.7%)
10. Immunological disorder	84 (46.4%)	37 (62.7%)	13 (61.9%)
11. Antinuclear antibody (ANA)^b^	178 (98.3%)	59 (100%)	21 (100%)

a Remission visit.

b Positive by immunofluorescence microscopy.

cSLEDAI, clinical SLEDAI-2K (excluding anti-dsDNA binding and complement consumption); HQ, hydroxychloroquine; PGA, physician’s global assessment of disease activity.

Serum from 181 incident and prevalent cases were selected for cross-sectional analysis. A subgroup of 21 patients was selected for longitudinal analysis based on the presence of fluctuating disease activity over time: *i.e.* ≥1 visit with observed high disease activity (flare), defined as an SLE disease activity index (SLEDAI-2K) score of ≥8 combined with a physician’s global assessment (PGA) score of ≥1 on a scale 0–4; and ≥1 visit without clinical disease activity (remission), with SLEDAI-2K score of ≤4 and PGA=0 (**Table 2**). For every patient, a flare visit was paired with the closest remission visit. Pharmacotherapy declared in tables (**Tables 1** and **2**) refers to drugs and doses prescribed after the visit to the rheumatologist.

**Table 2:** Detailed characteristics of the 21 patients with SLE selected for consecutive analyses.

Gender	cSLEDAI at flare visit	SLEDAI-2K at flare visit	PGA at flare visit	Prednisolone dose at flare visit* (mg/day)	DMARDs at flare visit*	Clinical manifestations at flare visit(SLEDAI-2K descriptors)	Months between flare and remission
F	8	10	2	30	Cyclo, HQ	Organic brain syndrome	2
F	8	10	1	10	MMF, HQ	Hematuria, proteinuria	12
F	8	12	1	15	AZA	Arthritis, proteinuria	9
F	8	10	1	10	AZA, HQ	Hematuria, pyuria	6
F	8	12	2	10	MMF	Hematuria, proteinuria	39
M	12	14	2	60	MMF, HQ	Urinary casts, hematuria, proteinuria	3
M	26	28	4	80	MMF	Organic brain syndrome, myositis, urinary casts, hematuria, proteinuria, rash	11
F	9	11	2	80	Rituximab, HQ	Lupus headache, fever	5
F	12	14	2	15	Rituximab, HQ	Urinary casts, hematuria, proteinuria	26
F	14	14	2	15	MMF, HQ	Lupus headache, arthritis, fever, leukopenia	52
F	8	10	2	7,5	AZA, HQ	Hematuria, proteinuria	2
F	16	20	3	60	Cyclo	Urinary casts, hematuria, proteinuria, pyuria	13
F	19	23	2	15	Belimumab, HQ	Urinary casts, hematuria, proteinuria, pyuria, pleurisy, fever	5
M	8	10	1	10	MMF	Proteinuria, rash, mucosal ulcers	4
F	12	14	2	10	MMF, HQ	Urinary casts, hematuria, proteinuria	42
F	10	14	1	10	MTX, HQ	Arthritis, hematuria, alopecia	17
M	16	16	2	40	MMF	Hematuria, proteinuria, urinary casts, pyuria	30
M	14	16	2	30	MMF	Urinary casts, proteinuria, pyuria, mucosal ulcers	3
F	16	18	3	30	MMF, HQ	Arthritis, urinary casts, proteinuria, pleurisy, pericarditis	8
F	12	12	2	30	Cyclo, HQ	Urinary casts, hematuria, proteinuria	11
F	18	22	3	30	Cyclo, HQ	Arthritis, urinary casts, hematuria, proteinuria, rash	2

AZA, azathioprine; cSLEDAI, clinical SLEDAI-2K; Cyclo, cyclophosphamide; DMARDs, disease modifying anti-rheumatic drugs; F, female; HQ, hydroxychloroquine; M, male; MMF, mycophenolate mofetil; MTX, methotrexate.

*Pharmacotherapy refers to drugs and doses prescribed after the visit to the rheumatologist.

Another group of SLE patients originated from a cross-sectional sub-cohort of the register designed to examine cardiovascular involvement (“SLE-VASK”) (23). These patients (*n*=59) had ≥1 year’s duration of SLE and available data on the IGS. Subjects in SLE-VASK were selected and stratified according to disease phenotypes; 1) skin & joint involvement, 95% females, mean age 42.9 years; 2) antiphospholipid syndrome (APS), 75% females, mean age 45.2 years; and 3) renal involvement, 89% females, mean age 41.2 years. None in the skin & joint group met the criteria for APS or lupus nephritis (22, 24). All cases with the APS phenotype fulfilled APS criteria (24). All patients with the renal phenotype had biopsy-proven lupus nephritis and fulfilled the renal ACR criterion (21), none of them were classified with APS. SLE-VASK patients donated peripheral blood separately from their regular visits and were considered to have a low/stable disease activity at the time of sampling. Mean time between sampling and disease activity assessment for these patients was 3.8 months (23).

As controls, we employed sera from 70 blood donors (89% females, mean age 48.9 years), stored at the Clinical Immunology & Transfusion unit, to match the cross-sectional SLE cohort regarding age and gender.

### Clinical Routine Assessments

Routine laboratory analyses and clinical assessment were performed at each visit for the 181 patients selected for cross-sectional analysis, as well as the 21 cases selected for consecutive analysis. Evaluation of SLE disease activity was performed by four consultant rheumatologists at the rheumatology unit, University Hospital in Linköping, according to the SLEDAI-2K and/or clinical SLEDAI, excluding scores for laboratory items (anti-dsDNA and low complement) (8, 25). PGA of disease activity was also recorded at each visit. Routine lab included blood cell counts, urine analysis, ESR, high sensitivity CRP, creatinine, creatine kinase, antinuclear antibody (ANA) fine-specificities using addressable laser bead immunoassay (ALBIA) and the FIDIS™ Connective profile, Solonium software ver. 1.7.1.0 (Theradiag, Croissy-Beaubourg, France), complement protein (C) 3, C4 and C3d, as well as classic complement function assessed by a haemolytic assay (26).

### Serum IFN-α and the Type I IFN Gene Signature

Serum levels of IFN-α was measured by dissociation-enhanced lanthanide fluoroimmunoassay (DELFIA) as described elsewhere (27). The sensitivity of the assay was at least 1 IU/mL.

The type I IFN gene signature (IGS), represented by IFI27, IFI44, IFI44L and RSAD2 (28), was measured by qPCR. Peripheral blood mononuclear cells (PBMCs) were isolated from heparinised whole blood by density gradient centrifugation, washed and lysed by RLT-buffer. Total RNA was extracted using the RNeasy Mini Kit (Qiagen, Germany). RNA quantity and purity were assessed on a DS-11 spectrophotometer (DeNovix Inc. Wilmington, DE, USA). Total RNA was reverse transcribed using iScript™ cDNA synthesis kit (Bio Rad, USA) to obtain cDNA for RT-qPCR. The RT-qPCR was carried out at following conditions, preheating for 10 min at 95°C, and then repeating 40 cycles of 95°C 15 sec and 60°C for 60 sec using a StepOnePlus (Applied Biosystems, CA, USA). TaqMan™ Gene Expression assay (FAM) (Applied Biosystems, CA, USA) was used according to the manufacturer’s instruction with the following primers (ThermoFisher scientific), IFI44L: Hs00199115_m1, RSAD2: Hs00369813_m1, IFI27: Hs00271467_m1, IFI44: Hs00197427_m1, GAPDH: Hs03929097_g1.

The expression fold‐change of each target gene was determined by the relative quantification method (ΔΔCt) after normalisation to the housekeeping gene GAPDH and in relation to a control sample (pooled sample from 4 healthy controls). The IGS score is expressed as mean of log-transformed fold change of the four type I IFN induced genes.

### *In Vitro* Stimulation and qPCR of PBMC

PBMCs, isolated from healthy donor whole blood, were used for stimulation experiments (*n*=7). The cells were incubated for 24 hours with type I IFN inducing ICs. The ICs were created by the addition of 5 μg/mL of small nuclear ribonucleoproteins (snRNP; RNP/Sm antigen, Arotec diagnostics, Wellington, New Zeeland) and 200 μg/mL of total IgG (enriched from a patient with SLE showing high levels of anti-U1 RNP antibodies) to the cell cultures. As positive and negative control, 1 μg/mL of CL097 (toll-like receptor 7/8 agonist, from Invivogen, Toulouse, France) or cell culture medium (mock) was used, respectively. Cell culture supernatants were collected after 24h incubation and analysed for cytokines, chemokines and galectin-9. In addition, gene expression analysis of IFIT3 and MXA was performed in cell lysates using qPCR to confirm type I IFN induction by the ICs.

Total RNA extraction from PBMCs was performed using the ISOLATE II RNA Mini Kit from Bioline (London, UK) and cDNA was synthesised by using the SuperScript III Reverse Transcriptase First Strand cDNA Synthesis kit (Invitrogen, Carlsbad, CA, USA). For quantitation of gene transcripts, the SensiFAST SYBR^®^ Hi-ROX Kit (Bioline) was used and analysed in a CFX96 Touch Real-Time system (BIO-RAD Inc.). In all runs mRNA transcripts were normalised against the two housekeeping genes actin and GAPDH according to the 2^-ΔΔCt^ method. Data was thereafter further normalized against the Mock value according to Rieu et al. to reduce inter plate variation (29).

### Galectin-9, Chemokines and Cytokines

A high sensitivity multiplex assay was used to quantify IL-10, IL-6, IL-1β and tumour necrosis factor (TNF) in consecutive samples (R&D systems, Abingdon, UK) and cross-sectional samples (Milliplex, Millipore, Solna, Sweden). In the 181 cross-sectional samples, IL-1ra was analysed using ELISA (R&D Systems, Abingdon, UK). The BD Cytometric Bead Array Human Inflammatory Cytokines Kit (BD Biosciences, San Jose, CA, USA) was used to measure TNF in supernatants. Galectin-9, chemokines and other cytokines (not mentioned above) was measured by magnetic Luminex assay (R&D systems). IL-10, IL-6, IL-1β and TNF was analysed in 149/181 (82%) of the cross-sectional samples.

### Statistical Analyses

None of the key variables (IGS score, IFN-α or SLEDAI-2K) were normally distributed and hence only non-parametric statistical tests were used. Spearman’s correlation was used for all correlation analyses, Mann–Whitney U-test or Wilcoxon matched-pairs signed rank test was performed when comparing two groups. Kruskal–Wallis (non-matched samples) or Friedman’s test (matched samples) with Dunn’s multiple comparisons was used for comparisons of 3 groups. A two-sided p-value of <0.05 was considered statistically significant. Adjustments for multiple testing were not performed.

### Ethical Considerations

This study was carried out in accordance with the Declaration of Helsinki. Written informed consent was obtained from all participants. The study protocol was approved by the Regional Ethics Review Board in Linköping, Sweden (Decision number M75-08).

## Results

### Galectin-9 and Chemokines Are Associated With the Type I IFN Gene Signature (IGS)

**Figure 2:**
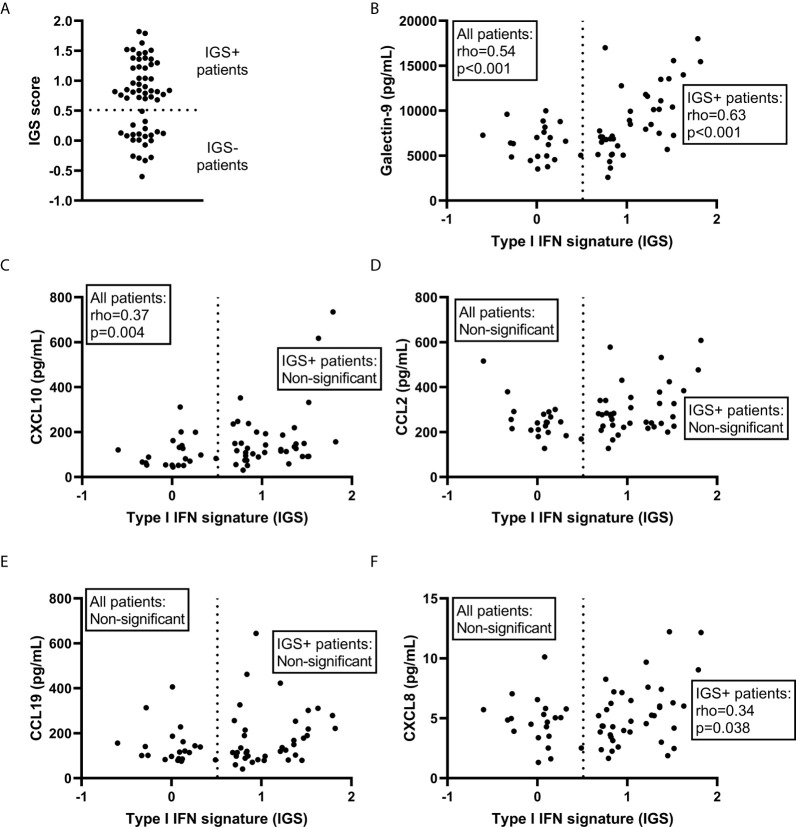
Correlations with the type I IFN gene signature (IGS). The IGS score of systemic lupus erythematosus patients (*n*=59) **(A)** and its correlation with galectin-9 **(B)** and selected chemokines **(C–F)** among all patients as well as among the IGS+ patients. Dashed line represents the cut-off for IGS positivity (IGS+).

The IGS score of the SLE-VASK cohort was significantly correlated with levels of galectin-9 (p<0.001, rho=0.54) and CXCL10 (p=0.004, rho=0.37), but not with CCL2, CCL19 or CXCL8 (**Figure 2**). Given the apparent distribution into two groups for the IGS, applying cut-off level at IGS score 0.5 (**Figure 2A**), correlation analyses were also performed among patients judged to be IGS positive (IGS score >0.5; *n*=38). This analysis revealed significant correlation between the IGS and galectin-9 (p<0.001, rho=0.629) and CXCL8 (p=0.038, rho=0.34), but not with CXCL10, CCL2 and CCL19 (**Figure 2**). To reveal potential differences between disease phenotypes, a correlation analysis of the IGS, galectin-9 and chemokines was also employed within every phenotype group (not shown). The IGS score of the APS phenotype group showed significant correlation only with CXCL10 (p=0.038; rho=0.47; *n*=20). Among patients with the renal phenotype, only galectin-9 was significantly correlated with the IGS score (p=0.006; rho=0.60; *n*=19). The IGS score of the skin & joint phenotype group was significantly correlated with galectin-9 (p=0.003; rho=0.62; *n*=20) as well as CXCL10 (p=0.011; rho=0.56; *n*=20).

In summary, galectin-9 was correlated with the IGS score in all subgroups except APS. CXCL10 was correlated with IGS in all subgroups except among those with renal involvement and the subgroup of IGS positive patients.

### Galectin-9 and Chemokines Are Associated With Circulating IFN-α Levels

**Figure 3:**
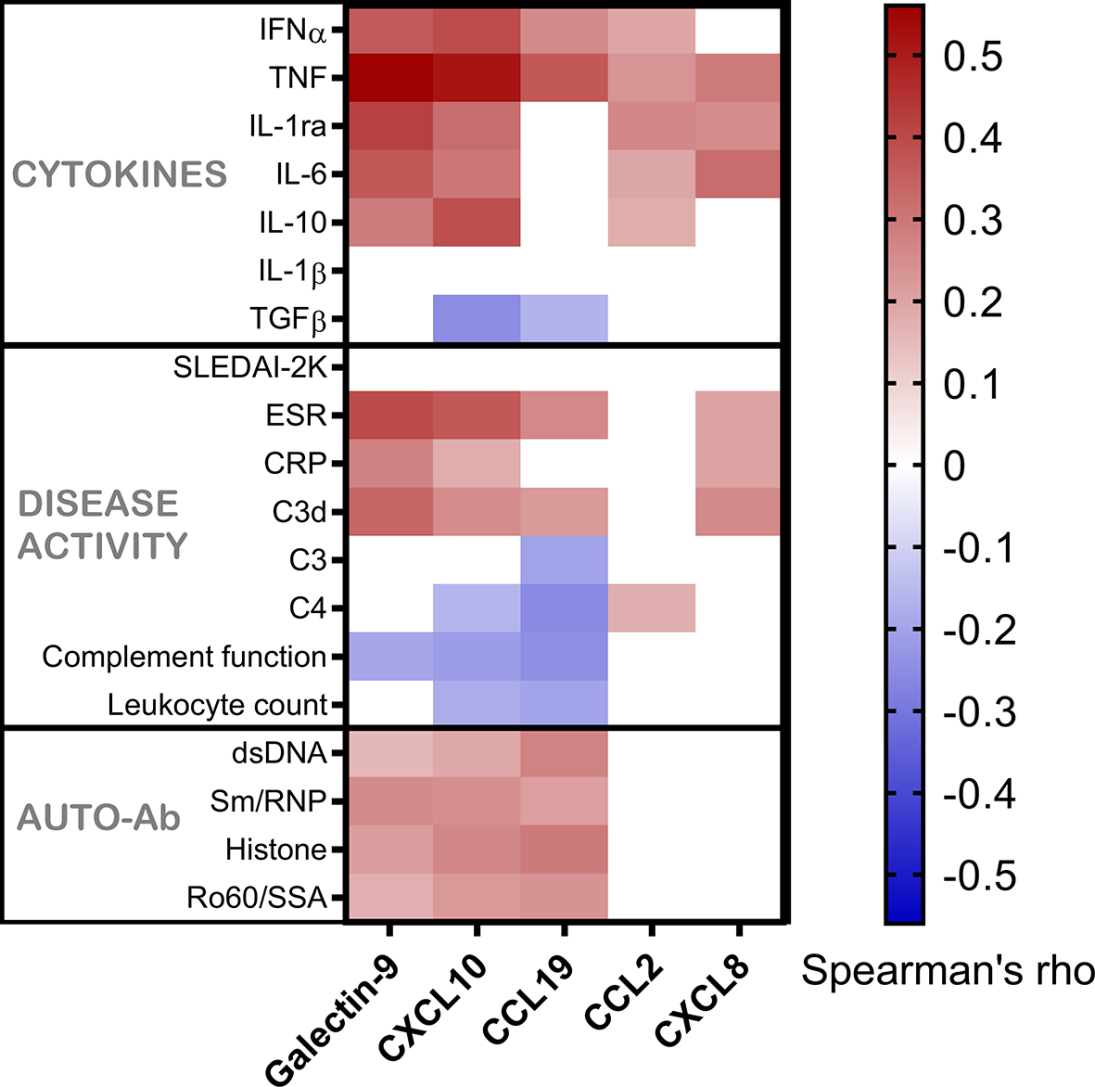
Galectin-9 and chemokines in relation to serum IFN-α, other cytokines, disease activity markers and autoantibodies. Serum levels of galectin-9 and chemokines in patients with SLE, shown as correlation with cytokines, disease activity variables and autoantibodies. Correlations are shown by a heat-mapping of Spearman’s correlation coefficient. White areas indicate a non-significant correlation (p > 0.05).

**Figure 4:**
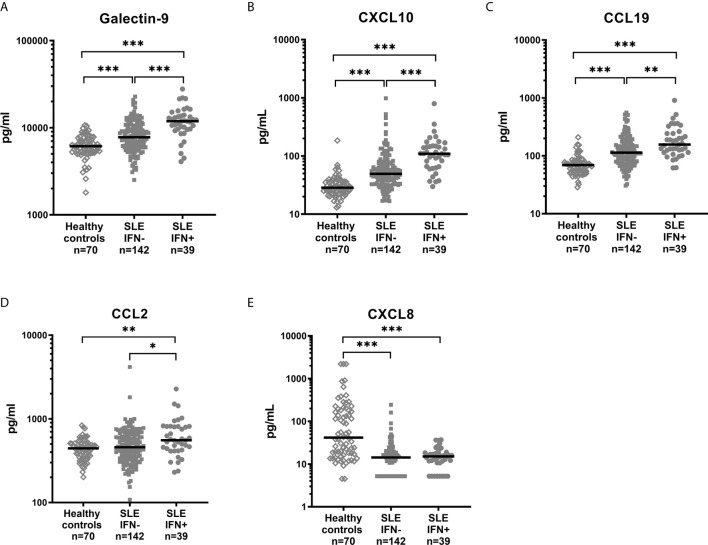
Levels of galectin-9 **(A)** and chemokines **(B–E)** in healthy controls and SLE patients stratified based on detectable serum IFN-α. Dots represent individual values and lines the median value. *p<0.05; **p<0.01; ***p<0.001.

To further clarify the associations, galectin-9, CXCL10, CCL19, CCL2, CXCL8 and serum levels of IFN-α were measured in the 181 cross-sectional samples. IFN-α was significantly correlated with galectin-9 (p<0.001, rho=0.36), CXCL10 (p<0.001, rho=0.39), CCL19 (p<0.001, rho=0.26) and CCL2 (p=0.009, rho=0.19), but not with CXCL8 (Spearman’s rho for correlations is illustrated in **Figure 3**). IFN-α was detected in 39 patients (21.5%) and when these patients were analysed separately no statistically significant correlation was found between IFN-α levels and galectin-9, or any of the chemokines (not shown). Stratification of all SLE patients based on the presence of detectable IFN-α, and comparisons with healthy controls (**Figure 4**) revealed a significant increase in levels of galectin-9 (p<0.001), CXCL10 (p<0.001), CCL19 (p=0.004), and CCL2 (p=0.019) but not CXCL8 among patients with detectable IFN-α (**Figure 4**). Healthy controls had significantly (p<0.001) lower levels of galectin-9, CXCL10 and CXCL19 compared with patients without detectable IFN-α, as well as patients with detectable IFN-α. CCL2 was significantly lower among healthy controls compared to patients with detectable IFN-α (p=0.002). Levels of CXCL8 were significantly higher (p<0.001) in healthy controls compared to patients regardless of IFN-α positivity (**Figure 4**).

In summary, serum IFN-α showed association with galectin-9, CXCL10, CCL19 and CCL2, but not with CXCL8.

### Galectin-9 and Chemokines Did Not Associate With SLEDAI-2K in Cross-Sectional Analysis

**Figure 5:**
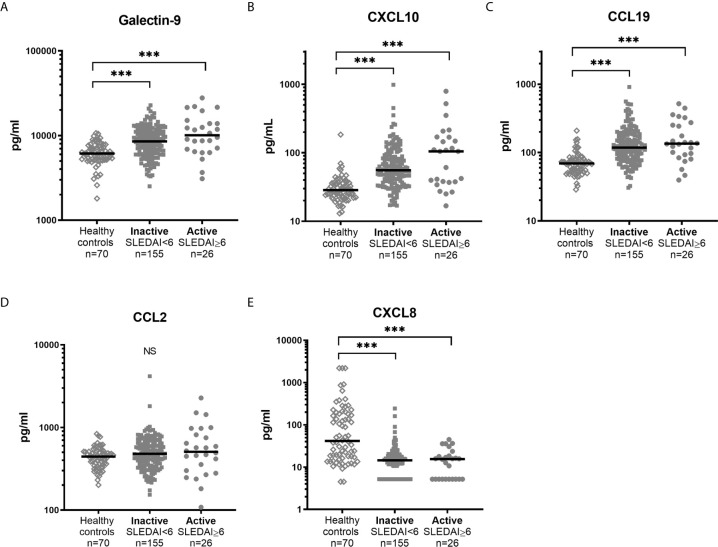
Levels of galectin-9 and chemokines in healthy controls and SLE patients with high and low disease activity. Galectin-9 **(A)** and chemokine levels **(B–E)** in sera from healthy controls and patients with SLE stratified based on disease activity (SLEDAI-2K). Dots represent individual values and lines the median value. ***p<0.001; NS, non-significant.

**Figure 6:**
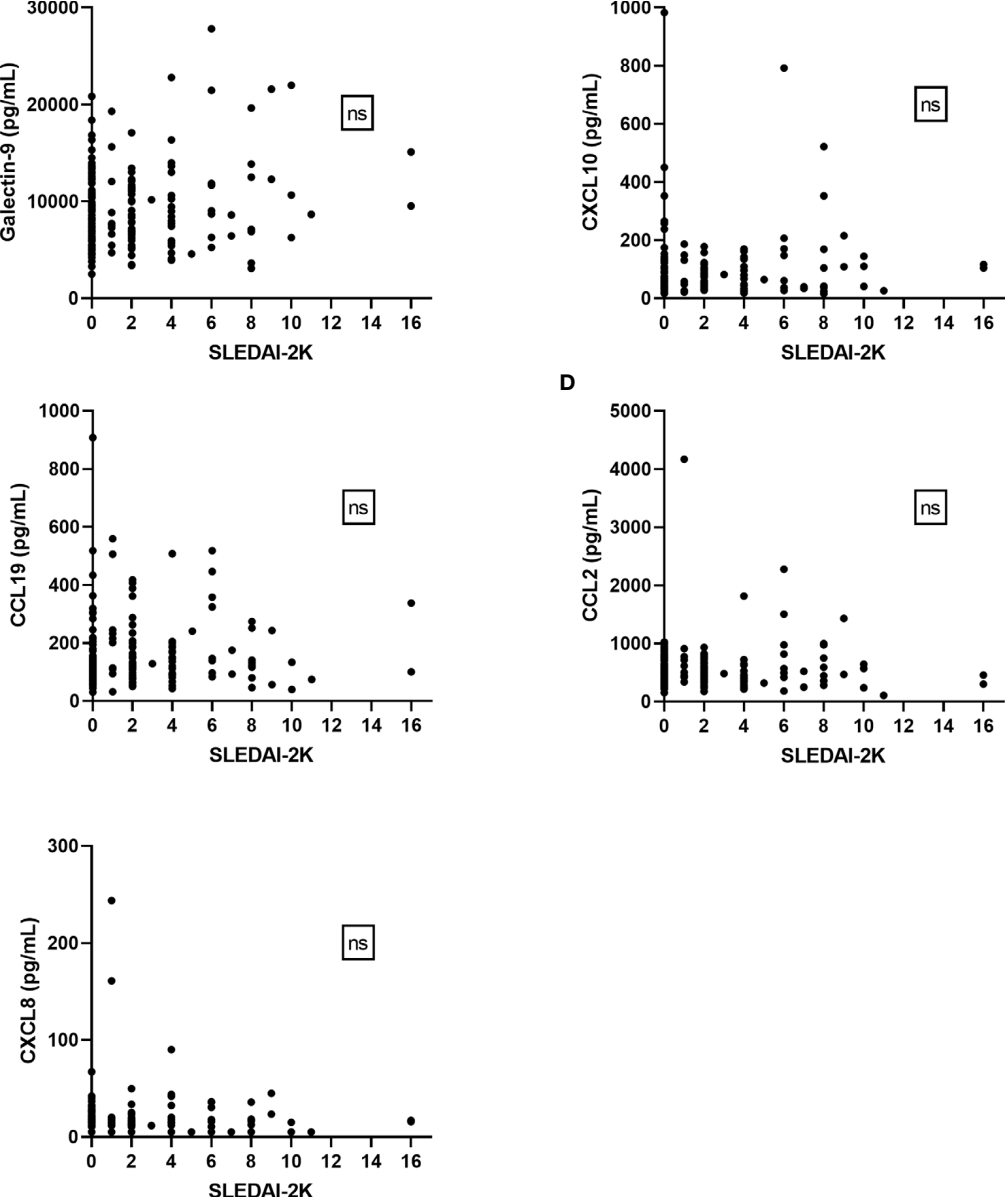
Galectin-9 and chemokines in relation to disease activity (SLEDAI-2K). No significant correlations were found between SLEDAI-2K and galectin-9 **(A)** or any of the chemokines **(B–E)** by Spearman’s correlation analysis. ns, non-significant.

Healthy controls had lower levels (p<0.001) of galectin-9, CXCL10 and CCL19 as compared to patient groups stratified according to SLEDAI-2K (**Figure 5**). CXCL8 levels were significantly higher among controls (p<0.001). No statistically significant difference between healthy controls, inactive patients (SLEDAI-2K<6) and active patients (SLEDAI-2K≥6) was found regarding CCL2. No significant differences in galectin-9 or any chemokines were found between active and inactive SLE patients (**Figure 5**). Correlation analysis revealed no statistical significances between SLEDAI-2K and galectin-9 or the chemokines (**Figure 6**). The correlation of galecin-9 and chemokines with cytokines, disease activity related measures and autoantibody levels are illustrated in **Figure 3**. In general, galectin-9 and CXCL10 showed stronger correlations with cytokines (including IFN-α), whereas CCL19 instead correlated inversely with complement levels/function and leukocyte count. CCL19, CXCL10 and galectin-9 were also correlated with autoantibody levels (**Figure 3**).

Importantly, we observed a statistically significant correlation between SLEDAI-2K and IFN-α (p=0.008, rho=0.198) among the 181 patients (**Supplementary Figure 1**). Furthermore, among IFN-α positive patients a significantly higher SLEDAI-2K was observed (median 2) compared to patients without detectable IFN-α (median 0; p=0.015).

SLE cases with secondary APS (*n*=32) had significantly higher levels of CXCL10 (median 72.8 pg/mL; p=0.007) compared to those without APS (*n*=149; median 53.5 pg/mL). Galectin-9, CCL2, CCL19 and CXCL8 levels did not differ significantly between patients with and without APS (not shown).

Taken together, despite their association with IFN-α, galecin-9 and chemokines were not associated with disease activity (**Figure 6**) but showed some correlations with laboratory variables of relevance for SLE pathogenesis (**Figure 3**).

### Galectin-9 Shows a Robust Association With TNF Which Is Independent of IFN-α

**Figure 7:**
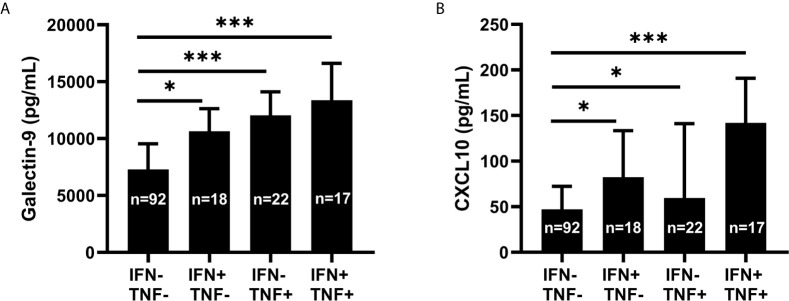
Galectin-9 and CXCL10 levels in relation to serum IFN-α and TNF. Serum galectin-9 **(A)** and CXCL10 **(B)** in patients with systemic lupus erythematosus (SLE), stratified based on serum interferon-α positivity (IFN+) and high levels of tumour necrosis factor (TNF+). Graphs show median (bars) with interquartile range (error bars). Kruskal Wallis test with Dunn’s multiple comparison. *p<0.05; ***p<0.001.

As the correlation between galectin-9 and TNF (p<0.001, rho=0.56) was the strongest correlation achieved among the tested variables (**Figure 3**), the interrelationship between TNF, IFN-α and galectin-9 was further investigated in detail. Since TNF, IFN-α and galectin-9 levels were all significantly correlated with each other (p<0.001), IFN-α and TNF were transformed to binary variables in further investigations. Detectable levels were considered positive for IFN-α whereas the 75^th^ percentile was used to create the cut-off for TNF. Galectin-9 levels were lowest in TNF and IFN-α double-negative patients and highest in double-positive patients, whereas patients with high TNF and non-detectable IFN-α had the second highest median levels (**Figure 7A**). CXCL10 was also strongly correlated with TNF (p<0.001, rho=0.514) and was similarly investigated. CXCL10 levels were lowest in double-negative patients and highest in double-positive patients, but in contrast to galectin-9, IFN-α positive patients without high TNF had the second highest median levels of CXCL10 (**Figure 7B**).

In short, circulating TNF levels showed a strong association with galectin-9. This association was present also in patients without detectable IFN-α.

### Galectin-9 and Chemokines in Consecutive Samples

**Figure 8:**
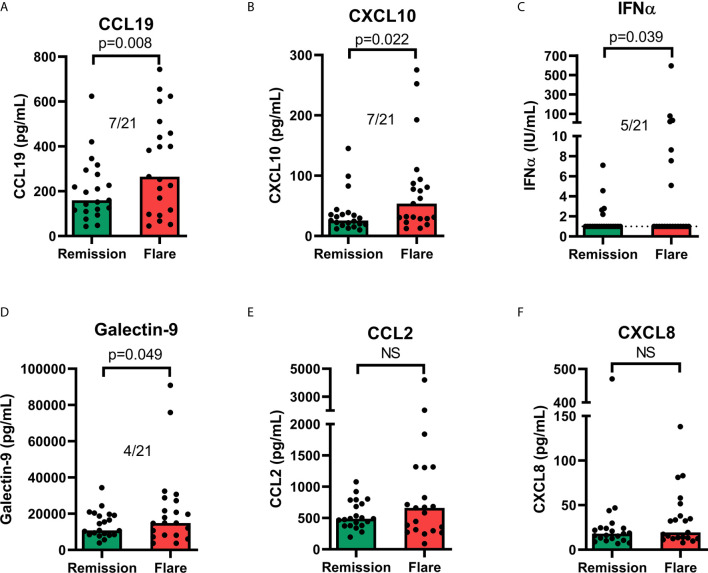
Serum levels of chemokines **(A, B, E, F)**, IFN-α **(C)** and galectin-9 **(D)** in remission versus flare. The graphs appear in the order of statistical significance (Wilcoxon matched-pairs signed rank test). The proportion of patients with an increase (≥2-fold) of the respective analyte between remission and flare is stated for significant results. Limit of quantitation (LOQ) is given as a dashed line where there are patient values below LOQ. Bars show median value. ns, non-significant.

In 21 patients (**Table 2**), galectin-9 and the chemokines were tested for their ability to reflect disease activity longitudinally at an individual level (**Figure 8**). Additional chemokines, cytokines and CRP were included in these analyses (**Supplementary Figure 2**). Pairwise comparisons revealed a statistically significant increase at flare compared to remission for IFN-α, galectin-9, CXCL10 and CCL19, but not for CCL2 or CXCL8 (**Figure 8**). Cytokine levels (IL-10, IL-1ra, IL-1β and TNF), CXCL11 and CXCL16 were also significantly increased in flare samples compared to remission samples (**Supplementary Figure 2**). Among the analytes reaching a statistically significance, the number of patients with ≥2-fold increase between remission and flare is stated in the graphs (**Figure 8** and **Supplementary Figure 2**). The purpose of this was to reveal any clinically meaningful change in biomarker levels. This resulted in a maximum of 8 out of 20 patients having ≥2-fold increase in IL-10, followed by CCL19, CXCL11, CXCL10, IL-1ra and IL-1β (7/21), TNF (6/21), IFN-α (5/21) and galectin-9 (4/21).

To summarise, many analytes were significantly increased at flare compared to remission, but the increases were generally modest and only a few patients displayed a marked increase (≥2-fold) at flare.

### IFN-α Induction in PBMCs and Concurrent Production of Chemokines, Galectin-9 and TNF

An *in vitro* model system for IC-driven disease activity with type I IFN activation as well as non-IC-driven type I IFN activation was used to investigate induction of galectin-9, chemokines and TNF. Stimulation of healthy donor PBMCs with CL097 (a TLR-7/8 agonist) as well as snRNP-containing ICs resulted in activation of the type I IFN system as confirmed by MXA and IFIT3 mRNA expression (**Supplementary Figure 3**). Protein levels of galectin-9 was only significantly increased by CL097 (**Supplementary Figure 3A**). Significantly increased protein levels of CCL2, CXCL8 and TNF were found following IC stimulation but not CL097 stimulation (**Supplementary Figures 3C, D**). CXCL10 production was not induced by IC or CL097. In conclusion, the galectin-9 and cytokine responses differed depending on the type I IFN inducing stimuli.

## Discussion

As knowledge of the importance of IFN-α in SLE has evolved, great interest has emerged to quantify type I IFNs for clinical implementation and possibly with impact on future decision-making regarding IFN-α targeted therapies (9). The present study was primarily undertaken to evaluate galectin-9 and chemokines in relation to type I IFNs, and ability to monitor disease activity in SLE patients.

Our data did not support previous observations on the usefulness of galectin-9 for disease activity monitoring but confirmed its association with IGS. Both IFN-α serum levels and IGS were clearly reflected by galectin-9 as well as CXCL10. None of the chemokines, nor galectin-9, reflected disease activity in cross-sectional analysis. Importantly, galectin-9 and CXCL10 were both highly correlated with TNF, and the results imply that the correlation between galectin-9 and type I IFN might be dependent on the association between galectin-9 and TNF. Longitudinal analysis revealed CCL19 as the most promising SLE disease activity biomarker among the ones examined here.

We observed that levels of galectin-9 were moderately associated with the IGS score, with a stronger association among patients affected by skin, joint and renal involvement than in SLE with concomitant APS. The latter contrasts with the report by van den Hoogen et al. who found that galectin-9 reflected IGS equally well among SLE patients with and without APS (16). This could perhaps be related to differences in cell types and selected genes to generate the type I IFN gene score. Measurement of protein levels of IFN-α in serum is a less sensitive assay than the IGS, herein detecting IFN-α in only 22% of the SLE cases. This could possibly be explained by the fact that patients were well controlled and used antimalarial agents to a high extent (30). Still, and in line with the report by Matsuoka et al., levels of galectin-9, as well as CXCL10, CCL2, and CCL19, were significantly higher among those with detectable serum IFN-α than among the others.

No associations between SLEDAI-2K or any of the chemokines/galectin-9 was found in the present study. Instead, the strongest correlations were achieved between galectin-9 *vs*. TNF, and CXCL10 *vs*. TNF. In fact, galectin-9 appeared to reflect TNF levels to a higher extent than serum IFN-α.

TNF is a strong pro-inflammatory cytokine of importance in SLE, but it may have been neglected since use of TNF-inhibitors is considered inappropriate although indications of efficacy have been demonstrated (31–33). It is well-known that increased TNF levels associate with disease exacerbations in SLE and *in vitro* data support that hydroxychloroquine decreases TNF-signalling by targeting the endosomal NADPH oxidase (34, 35). Furthermore, TNF is released by intrinsic renal cells co-localised with tissue-bound ICs and TNF is richly abundant in renal biopsies of patients with proliferative nephritis (36–38). In support of our findings regarding TNF, the *in vitro* model using IC stimulation of PBMCs resulted in profound TNF production and such IC driven TNF production has been demonstrated previously (39). Of further interest in this context, two recent studies have reported galectin-9 as a valuable biomarker of disease activity in another TNF-driven disease – namely rheumatoid arthritis (RA) (40, 41).

Studies on the functional role of galectin-9 in human SLE are lacking, and data from lupus mouse models show divergent results. Supplementation of galectin-9 decreased the disease severity in MRL/lpr, NZB/W-F1 and BXSB/MpJ, possibly *via* inhibition of toll-like receptors 7/9-induced IFN production by plasmacytoid dendritic cells (42, 43). On the other hand, galectin-9 gene -/- in BALB/c mice (pristane-induced) showed a less severe lupus-like disease (44).

Our *in vitro* experiments revealed different responses of IC induced versus TLR7/8-induced production of cytokines and galectin-9. This may indicate a complexity of IC induced responses in a mixed cell culture that, on one hand, activates TLR-7 and type I IFN regulated genes (30), but on the other hand stimulates chemokine production independent of TLR-7/8 activation. Another explanation could be that TLR7/8 activation results in different inflammatory profiles depending on the agonist, previously shown by Berggren et al. (39). Herein, galectin-9 was induced by CL097 but not by the ICs. As SLE disease activity is thought to be driven by ICs in many patients, the lack of IC-dependent galectin-9 induction could potentially explain the lack of correlation between galectin-9 and SLEDAI-2K in the clinical samples. Such conclusion is however contradicted by the lack of correlation between SLEDAI-2K and CCL2 or CXCL8 despite the distinct IC-dependent induction of these chemokines *in vitro*.

The present study’s analyses of longitudinal samples did not reveal any biomarker with potential to be used in clinical routine to monitor patients over time. Among the chemokines, a maximum of 7 out of 21 patients had a pronounced (>2-fold) increase in biomarker level at flare (CCL19, CXCL11 and CXCL10). Galectin-9 levels were increased (>2-fold) at flare only among 4 of the 21 patients.

Several reasons should be considered regarding the divergent results achieved herein compared to prior observations regarding associations of disease activity with CXCL8 (10) and galectin-9 (16). Ethnicity is closely related to genetics, severity of SLE and the type I IFN response (45). In addition, both the overall disease activity and the distribution of SLE phenotypes in the study populations are of relevance since certain manifestations and disease mechanisms may be more strongly linked to activation of type I IFNs than others. For instance, in our study, 20-30% had lupus nephritis whereas this proportion may differ significantly with regard to both patient selection and ethnicity. Our study included a large number of patients compared to similar reports, and the disease manifestations are described in **Tables 1** and **2**.

The limitations of this study include a small number of patients of non-Caucasian ethnicity and relatively few patients with ‘high’ (PGA=3) to ‘very high’ (PGA=4) disease activity. The latter may probably be a result of generally well-controlled patients and a public and tax-funded health care system with universal access. The use of large cohorts with well-characterised patients followed by a limited number of experienced rheumatologists (*n*=4) at a single tertiary referral centre constitutes a major strength of this study.

To summarise, clinical follow-up of patients with SLE remains challenging. In line with previous reports, we found that galectin-9 reflected both the IGS and serum IFN-α. However, longitudinal associations between galectin-9 and clinical or laboratory SLE disease activity measures were not clear-cut and CXCL10 as well as CCL19 performed better than galectin-9 in this regard. In our hands, galectin-9 showed more convincing associations with TNF, a cytokine with established importance in both RA and SLE, than with IFN-α. Our results indicate that, despite associations with IFN-α, surrogate biomarkers of type I IFNs may be influenced by many other factors and is therefore not necessarily useful in clinical practice for surveillance of lupus disease activity.

## Data Availability Statement

The raw data supporting the conclusions of this article will be made available by the authors, without undue reservation.

## Ethics Statement

The studies involving human participants were reviewed and approved by the Regional Ethics Review Board in Linköping, Sweden. The patients/participants provided their written informed consent to participate in this study.

## Author Contributions

HE, JW, and CSj conceived the original idea and project planning. HE, JW, BG, AAB, LR, and CSj contributed to the study design. CSj collected clinical patient data. HE, BG, M-LE, and CSv carried out the laboratory work. HE, JW, M-LE, BG, CSv, ML, and CSj analysed the data. HE, JW, ML, and CSj drafted the manuscript. All authors contributed to the article and approved the submitted version.

## Funding

This work was supported by the Swedish Rheumatism association (R–844801); the Region Östergötland ALF Grants (LIO–791961); the King Gustaf V’s 80-year Anniversary Foundation (FAI–2018–0504 to CSj); the King Gustaf V, Queen Victoria’s Foundation of Freemasons; the Alfred Österlund’s Foundation; the Anna-Greta Crafoord Foundation; the Greta and Johan Kock’s Foundation; the Skåne University Hospital; the Medical Faculty of Lund University; the Swedish Research Council for Medicine and Health (2018–02399, 2018-02516 and 2017-01091) and the Swedish Society of Medicine (the Ingegerd Johansson donation). 

## Conflict of Interest

The authors declare that the research was conducted in the absence of any commercial or financial relationships that could be construed as a potential conflict of interest.
