# Supplementary material for: Comparison of Surrogate Markers of the Type I Interferon Response and Their Ability to Mirror Disease Activity in Systemic Lupus Erythematosus
Source: Front Immunol. 2021 Jun 30;12:688753. doi: 10.3389/fimmu.2021.688753 (PMC8278235; doi:10.3389/fimmu.2021.688753)
Supplement: Supplementary file 1 [file DataSheet_1.pdf]

SUPPLEMENTARY FIGURE 1

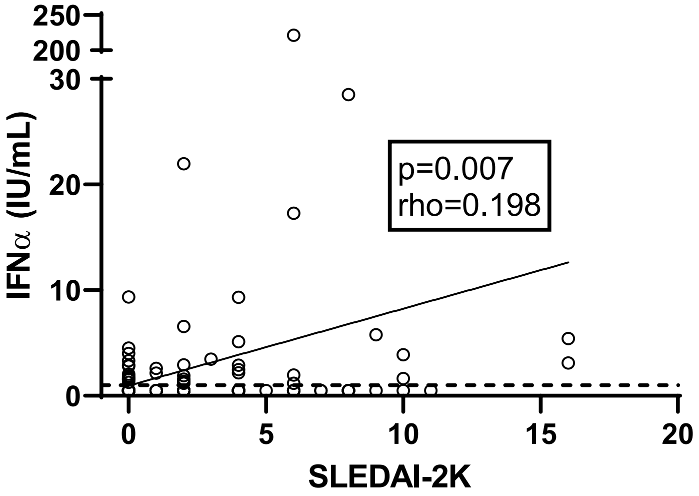

Supplementary Figure 1. Correlation between serum IFNα and disease activity. Circulating IFN-α and its correlation with disease activity (as defined by SLEDAI-2K) among 181 SLE patients selected for cross-sectional analysis. Circles below the dashed line represent serum samples where IFN-α was undetectable (<1 IU/mL).
