# Supplementary material for: Comparison of Surrogate Markers of the Type I Interferon Response and Their Ability to Mirror Disease Activity in Systemic Lupus Erythematosus
Source: Front Immunol. 2021 Jun 30;12:688753. doi: 10.3389/fimmu.2021.688753 (PMC8278235; doi:10.3389/fimmu.2021.688753)
Supplement: Supplementary file 2 [file DataSheet_2.pdf]

SUPPLEMENTARY FIGURE 2

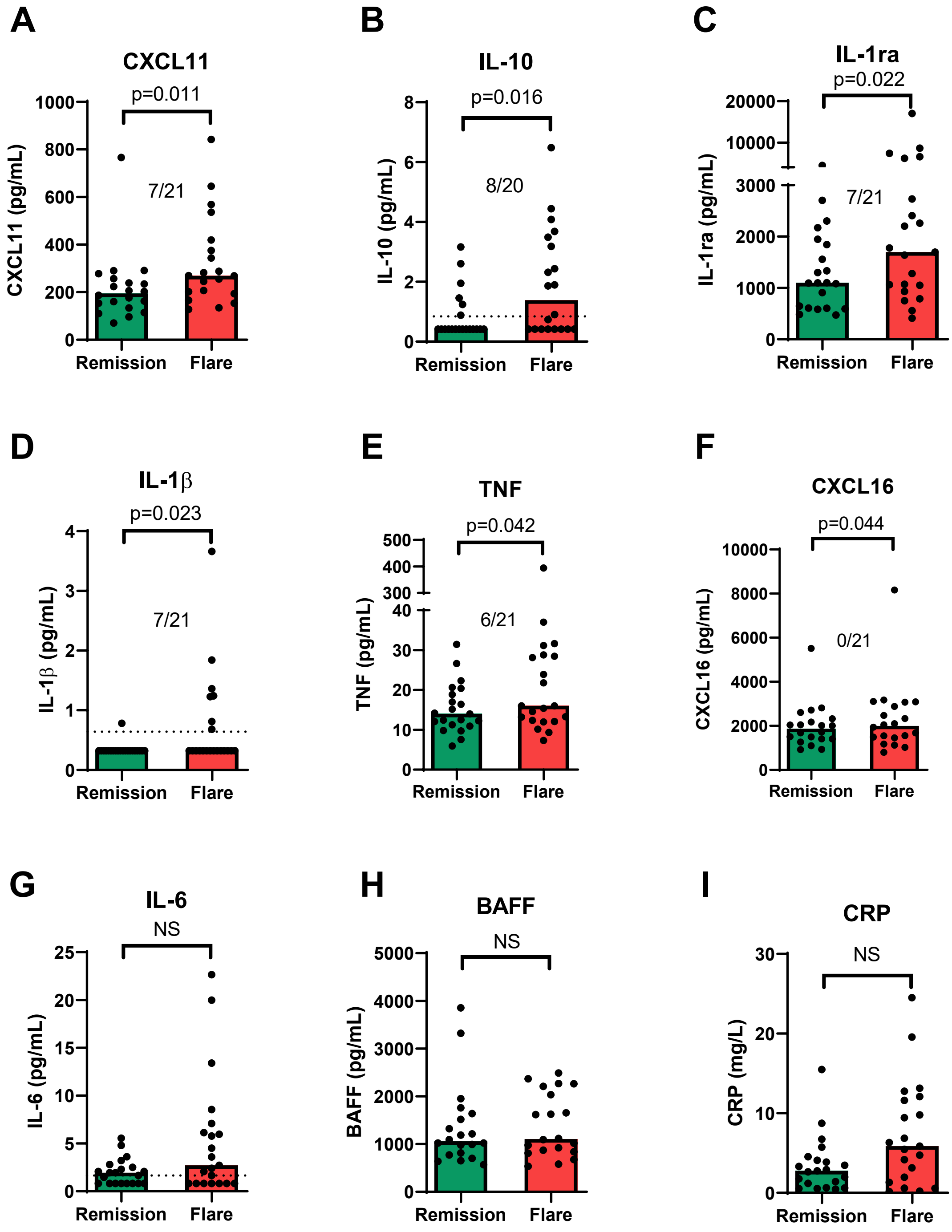

Supplementary Figure 2. Inflammatory markers in paired samples of remission and flare. Levels of chemokines (A and F), cytokines (B-E, G and H) and CRP (I) in patients followed consecutively. The graphs appear in the order of statistical significance (Wilcoxon matched-pairs signed rank test). The proportion of patients with an increase (≥2-fold) of the respective analyte between remission and flare is stated for significant results. Limit of quantitation (LOQ) is given as a dashed line in graphs where there are patient values below LOQ. Bars show median value.
