# Supplementary material for: Comparison of Surrogate Markers of the Type I Interferon Response and Their Ability to Mirror Disease Activity in Systemic Lupus Erythematosus
Source: Front Immunol. 2021 Jun 30;12:688753. doi: 10.3389/fimmu.2021.688753 (PMC8278235; doi:10.3389/fimmu.2021.688753)
Supplement: Supplementary file 3 [file DataSheet_3.pdf]

SUPPLEMENTARY FIGURE 3

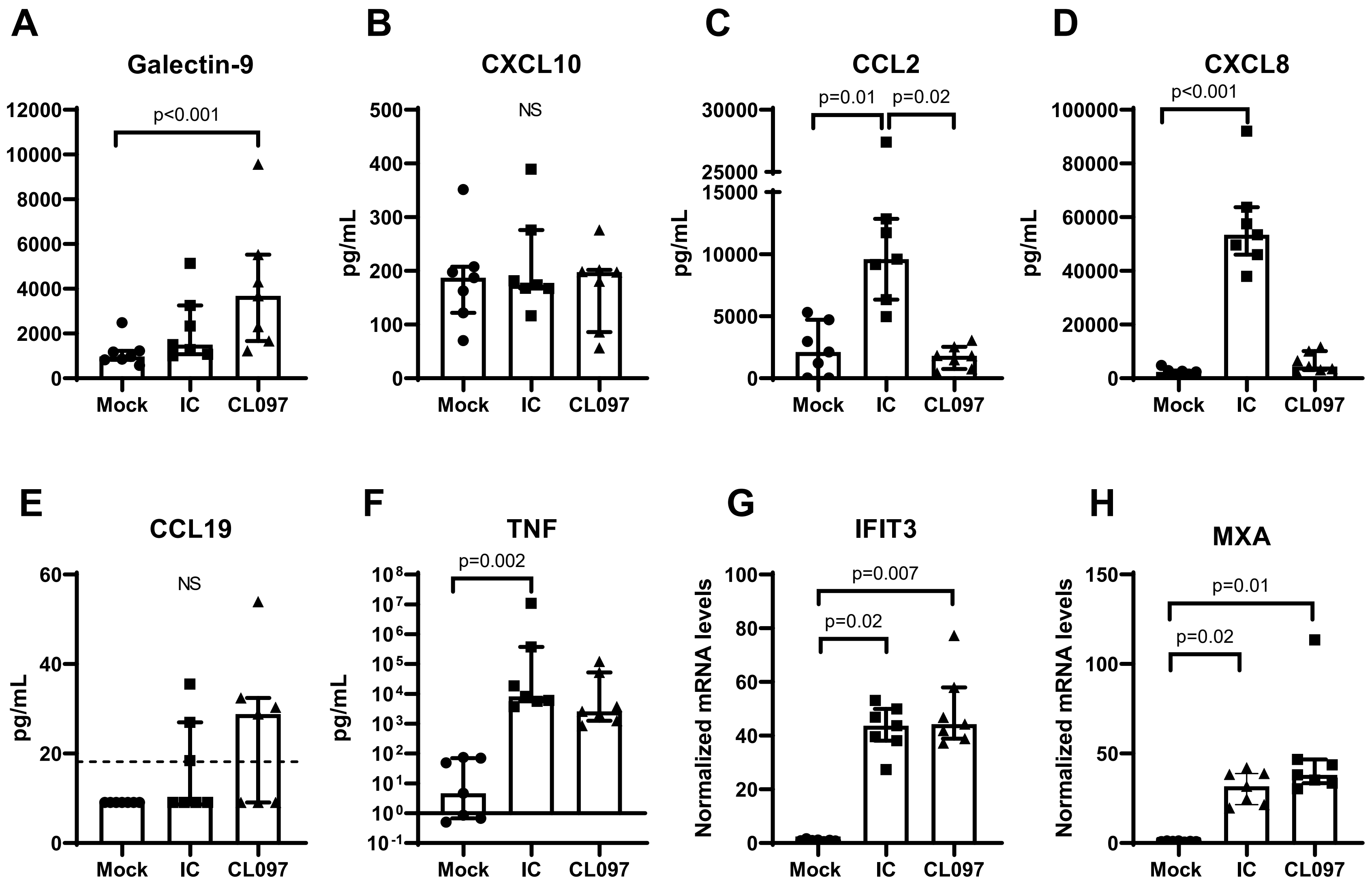

Supplementary figure 3. Production of galectin-9, TNF and chemokines from IC stimulated and TLR7/8 activated cells. Peripheral blood mononuclear cells of healthy donors were stimulated with immune-complexes (IC; *i.e.* snRNP + IgG) or CL097 (TLR7/8 agonist) to induce type I IFN activation. Production of galectin-9, chemokines and TNF was measured (A–F) and the activation of type I IFN stimulated genes was confirmed by qPCR analysis of IFIT3 and MXA (G-H). Graphs show median (bars) with IQR (error bars). Friedman's test with Dunn's multiple comparisons was performed to compare levels of galectin-9, chemokines and gene expression (normalized to Mock). A majority of the samples did not reach the limit of quantification for CCL19 (dashed line).
